# Healthcare for People With Diabetes in Pregnancy: A National Survey Comparing Metropolitan and Rural Care Delivery in Australia

**DOI:** 10.1111/ajo.70109

**Published:** 2026-03-25

**Authors:** Ellen Payne, Susan Heaney, Clare Collins, Megan Rollo, Leanne J. Brown

**Affiliations:** ^1^ University of Newcastle Department of Rural Health, College of Health, Medicine and Wellbeing, University of Newcastle Tamworth New South Wales Australia; ^2^ University of Newcastle Department of Rural Health, College of Health, Medicine and Wellbeing, University of Newcastle Port Macquarie New South Wales Australia; ^3^ Nutrition and Dietetics, School of Health Sciences College of Health, Medicine and Wellbeing, The University of Newcastle Callaghan New South Wales Australia; ^4^ School of Population Health, Faculty of Health Sciences Curtin University Bentley Western Australia Australia

**Keywords:** delivery of health care, gestational diabetes, health personnel, pregnancy in diabetics, rural health

## Abstract

**Background:**

Management of diabetes in pregnancy requires input from multiple health professionals throughout the course of an individual's pregnancy. Implementing healthcare delivery that suits the local context of a metropolitan or rural region may contribute to improvements in care delivery and pregnancy and birthing outcomes.

**Aims:**

To compare healthcare delivery for people with diabetes in pregnancy in metropolitan and rural areas of Australia.

**Materials and Methods:**

A cross‐sectional survey was conducted, with questions focused on healthcare delivery for women with diabetes in pregnancy, including the multidisciplinary care provided. Data were collected via a nationwide survey of health professionals currently involved in the healthcare management of people with diabetes in pregnancy in Australia. Survey data were analysed using descriptive and inferential statistics.

**Results:**

The main aspects of healthcare delivery were similar between metropolitan and rural respondents. The proportion of health professionals offering face‐to‐face services was greater in rural areas (*n* = 38, 100%) compared to metropolitan (*n* = 34, 71%). Rural respondents (21%) reported they were collocated with an endocrinologist and 48% with an obstetrician. This was compared to 71% of metropolitan respondents reporting being collocated with an endocrinologist, and 63% with an obstetrician.

**Conclusions:**

This research offers direction as to potential considerations when planning and implementing models of care in rural areas. Additional research confirming the priorities within the rural context is needed to support the development of optimal care delivery for pregnant people with diabetes living in rural regions.

## 1 Introduction

Diabetes in pregnancy (DIP) is an overarching term used to describe the existence of gestational diabetes mellitus (GDM) or pre‐existing type 1 or type 2 diabetes mellitus during a pregnancy. Individuals with DIP require assessment from a range of health professionals, and frequent monitoring is recommended for effective management and optimal birth outcomes [1, 2]. Management for people with all types of DIP involves dietary advice alongside self‐monitoring of blood glucose levels, with the use of medication when required [1, 2]. Individuals with pre‐existing DIP require monitoring throughout the entire pregnancy, with regular insulin titration required for those with type 1 diabetes mellitus and medication adjustments for people with type 2 diabetes mellitus [2]. Recommendations state monitoring of women with DIP should occur every 1 to 2 weeks, depending on the type of diabetes and clinical need [1, 2]. The health professional team for individuals with DIP frequently includes diabetes educators, dietitians, endocrinologists, obstetricians and midwives [3].

In the current Australian rural context, healthcare may be modified to suit the needs and resources of the region [4]. Adequate staffing is one key issue affecting healthcare delivery in rural Australia, with the number of health and medical specialists generally decreasing as location remoteness increases [5]. The geographic isolation and limited infrastructure in rural compared to metropolitan areas are additional barriers to healthcare access [5]. Developing healthcare services that address these unique challenges associated with rural healthcare delivery have been identified as key areas of improvement [4]. Despite this, research into effective healthcare delivery for the rural context remains limited [6, 7].

A model of care refers to how healthcare is delivered to manage a specific health condition [8]. In rural areas consideration of the model of care may be needed to optimise care provided, given the challenges of access, staffing levels and infrastructure [4]. Key elements for success include innovations in care, efficient service delivery, effective use of available resources and provision of patient‐centred care [4, 8, 9]. An Australian study describing 15 GDM care models reported offering initial face‐to‐face dietitian appointments; however, frequency of follow up, medication initiation and the health professionals responsible for ongoing insulin titration varied between clinics [10]. This research provides some insight into care delivery for GDM [10], however, a broader understanding of the care delivery needs in rural Australia is needed.

Of the several surveys of health professionals providing services for people with DIP [3, 11, 12, 13], Meloncelli et al. surveyed health professionals treating people with GDM [3] and their managers [11] about their current care practices and understanding of the different roles within the multidisciplinary team [3, 11]. Certain aspects of care delivery were perceived as the role of a particular professional, such as nutrition advice being the role of the dietitian, whereas checking blood glucose levels was reported to be the role of any team member by the majority of respondents [3]. However, there was no comparison made between the metropolitan and rural data [3, 11]. While these studies provided insights into aspects of healthcare delivery for people with DIP in Australia [3, 11, 12, 13], healthcare delivery for people with DIP in rural areas is an under‐researched topic which requires further exploration. Hence, the overall aim of the current study was to compare aspects of care delivery for people with DIP in metropolitan and rural areas of Australia.

## 2 Materials and Methods

A cross‐sectional survey of health professionals involved in the management of people with DIP across Australia was undertaken over 10 months in 2022. Survey questions were developed using Meloncelli's survey as an initial guide [3]. Respondents were asked to provide the street name and postcode of their primary place of work. Following a literature review to establish important aspects of healthcare delivery, two questions were modified and 20 added. Added questions focussed on communication processes of the multidisciplinary team, the impact of COVID‐19 on care and specific aspects as well as overall satisfaction with the model of care used. A Likert scale was used for questions focussed on ‘levels of satisfaction’. The survey was developed using REDCap [14, 15], a secure web‐based program for building and managing online surveys. The survey was reviewed and piloted by three health professionals with experience in the field who were not currently eligible for participation to establish face and content validity (Appendix S1).

Ethics approval was received from the University of Newcastle Human Research Ethics Committee (H‐2021‐0425). Informed consent was obtained through the completion and submission of the survey, as explained to participants in the participant information statement. Additionally, participants were informed that their data would only be used if they pressed ‘submit’ at the completion of the survey. To be eligible, health professionals needed to be practising in Australia and currently involved in the management of people with DIP. Eligible health professionals included but were not limited to dietitians, diabetes educators/diabetes nurse practitioners, antenatal nurses, midwives, endocrinologists, obstetricians and general practitioners.

Invitations to participate in the survey were disseminated via email through various professional organisations and membership bodies. Organisations were selected to target a wide range of health professionals who are involved in the care delivery of people with DIP. In addition to including national professional organisations, several rural organisations were targeted to optimise rural health professional recruitment. A total of 10 organisations were requested to disseminate the survey to their members; organisation details are provided in the results. Organisations were requested to email up to two reminders to their members and were also provided the option to promote the survey via their social networking platforms. A survey link was displayed at two relevant conference presentations. The survey was open from March 2022 to December 2022.

Quantitative responses were analysed in Stata IC‐16 (StataCorp, 2016) using descriptive statistics. The Modified Monash Model (MMM) was used to categorise the location of respondents [16] and converted to MMM categories 1–7, MMM1 was considered metropolitan, MMM2 a regional centre, MMM3–5 rural and MMM6–7 remote [16]. For analysis these were collated into metropolitan (MMM1) and rural (MMM2–7) [16]. Metropolitan and rural responses regarding satisfaction with the model of care and multidisciplinary team communication were dichotomised into ‘satisfied’ and ‘unsatisfied’. These were then compared using a chi‐squared test of independence after ensuring the appropriateness of this test based on cell count [17].

## 3 Results

The following organisations emailed their members the survey: Dietitian's Australia, Australian Diabetes Educator's Association, Rural Doctor's Network, Endocrine Society of Australia, Services for Australian Rural and Remote Allied Health (SARRAH), Dietitian Connection, Australasian Diabetes in Pregnancy Society (ADIPS), National Rural Health Alliance and 22 of Australia's 31 Primary Health Networks. Additionally, the survey was not disseminated by The Royal Australian and New Zealand College of Obstetricians and Gynaecologists and the remaining 9 of 31 Primary Health Networks.

One hundred and fifty people responded to the survey, with three respondents ineligible to participate. Of the 147 eligible respondents, 61 did not complete the entire survey, resulting in a total of 86 completed surveys. A response rate was unable to be calculated, as the total number of eligible participants reached via the survey's dissemination methods is unknown.

A total of 48 respondents worked in a metropolitan setting, with 38 working in a regional, rural or remote setting. Of those working in a non‐metropolitan setting, 11 worked regionally (MMM2), 20 worked rurally (MMM3–5) and 7 worked in a remote setting (MMM6–7) [16]. The majority of respondents were dietitians (*n* = 51, 54%), followed by diabetes educators (*n* = 25, 27%) and endocrinologists (*n* = 12, 13%). The most common work settings were public hospitals (*n* = 53, 52%), private practice (*n* = 19, 19%) and community health centres (*n* = 13, 13%). Participants could select multiple professions and work settings if this applied to them. See Table 1 for a complete summary of the demographic data.

**TABLE 1:** Health professional survey respondent demographics and role in the management of diabetes in pregnancy (*n* = 86).

	Metropolitan	Rural	Total
**State**			
New South Wales	19 (22%)	13 (15%)	32 (37%)
Queensland	6 (7%)	11 (12%)	17 (19%)
Western Australia	13 (15%)	2 (2%)	15 (17%)
Victoria	6 (7%)	6 (7%)	12 (14%)
Tasmania	0 (0%)	3 (3%)	3 (3%)
Northern Territory	0 (0%)	3 (3%)	3 (3%)
South Australia	2 (2%)	0 (0%)	2 (2%)
Australian Capital Territory	2 (2%)	0 (0%)	2 (2%)
**Health professional** ^**a**^			
Dietitian	25 (26%)	26 (27%)	51 (54%)
Diabetes educator/nurse/nurse practitioner	10 (10%)	13 (13%)	25 (26%)
Endocrinologist	11 (11%)	0 (0%)	11 (12%)
Obstetrician	2 (2%)	0 (0%)	2 (2%)
General Practitioner	1 (1%)	1 (1%)	2 (2%)
Midwife	1 (1%)	1 (1%)	2 (2%)
			Total: 94
**Work setting** ^**a**^			
Public hospital with specialised diabetes in pregnancy services	27 (26%)	10 (9%)	37 (36%)
Public hospital without specialised diabetes in pregnancy services	4 (4%)	12 (11%)	16 (15%)
Public diabetes centre	3 (3%)	3 (3%)	6 (5%)
Private hospital	6 (5%)	1 (1%)	7 (6%)
Private practice	14 (13%)	5 (5%)	19 (18%)
Community health centre	4 (4%)	9 (8%)	13 (12%)
Other	1 (1%)	2 (2%)	3 (3%)
			Total: 101
**Years worked in profession**			
< 1 year	1 (1%)	2 (2%)	3 (3%)
1–4 years	5 (6%)	6 (7%)	11 (13%)
5–10 years	11 (13%)	15 (17%)	26 (30%)
11–20 years	14 (16%)	9 (10%)	23 (27%)
21–30 years	8 (9%)	4 (5%)	12 (14%)
30+ years	9 (10%)	2 (2%)	11 (13%)
**Women with DIP seen each month**			
5 or less	4 (4%)	16 (18%)	20 (23%)
6 to 10	8 (9%)	7 (8%)	15 (17%)
11 to 20	3 (3%)	6 (7%)	9 (10%)
More than 20	33 (38%)	9 (10%)	42 (48%)
**Number of Years working in DIP**			
< 1 year	4 (4%)	1 (1%)	5 (5%)
1–4 years	14 (16%)	12 (14%)	26 (30%)
5–10 years	8 (9%)	14 (16%)	22 (25%)
11–20 years	17 (19%)	8 (9%)	25 (29%)
21–30 years	5 (5%)	1 (1%)	6 (7%)
> 30 years	0 (0%)	2 (2%)	2 (2%)

Abbreviation: DIP, diabetes in pregnancy.

^a^ Multiple responses possible.

**FIGURE 1:**
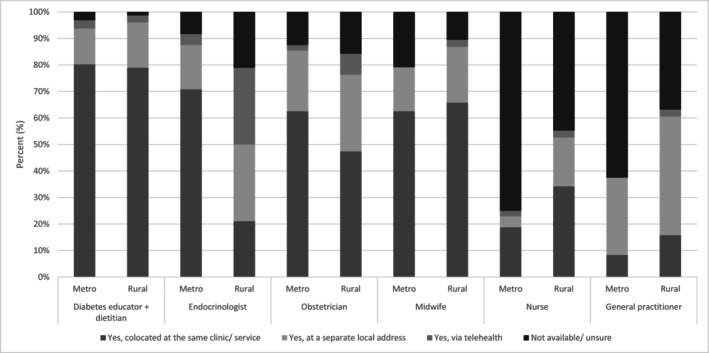
Multidisciplinary team members, co‐location and availability by location (rural vs. metropolitan). Survey question: Please indicate which members of the multidisciplinary team (MDT) are available in your clinic/service for women with diabetes in pregnancy (select all that apply).

Diabetes educators and dietitians were most frequently co‐located in the same clinic (*n* = 69, 80.2%, and *n* = 68, 79.1% respectively). Endocrinologists and obstetricians were less likely to be co‐located in rural areas compared to metropolitan, with 70.8% (*n* = 34) of metropolitan respondents co‐located with an endocrinologist, and 62.5% (*n* = 30) co‐located with an obstetrician, compared to 21% (*n* = 8) of rural respondents co‐located with an endocrinologist and 47.4% (*n* = 18) co‐located with an obstetrician. More information on health professional colocation can be seen in Figure 1.

**FIGURE 2:**
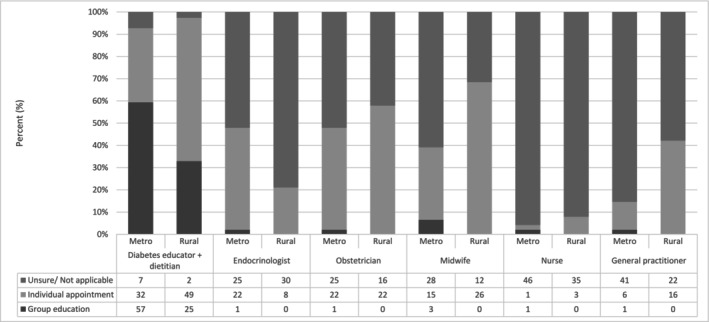
Delivery of initial education by health profession and location (rural vs. metropolitan). Survey question: After diagnosis, which health care professional(s) do women see for initial education and advice on managing gestational diabetes mellitus and what format does this take? (select all that apply).

Diabetes educators and dietitians were reported to primarily provide initial DIP education in a group format, as seen in Figure 2 (*n* = 43, 50%, and *n* = 39, 45.3% respectively). Metropolitan based diabetes educators and dietitians were more likely to provide group education (*n* = 30, 62.5% diabetes educators and *n* = 27, 56.3% dietitians) compared to those located rurally (*n* = 13, 34.2% diabetes educators and *n* = 12, 31.6% dietitians). (Chi‐squared test of independence: metropolitan vs. rural diabetes educators **p = 0.006; metropolitan vs. rural dietitian **p = 0.007).

All rural based health professionals offered initial education services face‐to‐face (100%, *n* = 38), compared to 71% (*n* = 34) of metropolitan based health professionals. The use of telehealth for initial patient education was similar between metropolitan (73%, *n* = 35) and rural (74%, *n* = 28) respondents. Table 2 provides a summary of the various modes of delivery used for initial education sessions with those who have GDM.

**TABLE 2:** Communication methods used by health professionals during care delivery and within multi‐disciplinary teams by location.

	Metropolitan *n* = 48, *n* (%)	Rural *n* = 38, *n* (%)	*p*
**Mode of initial education used** ^**a**^			
Face‐to‐face^c^	34 (70.8%)	38 (100%)	0.0003***
Telehealth^c^	35 (72.9%)	28 (73.7%)	0.94
Written information via post, email or fax^c^	19 (39.6%)	12 (31.6%)	0.44
Video presentation^c^	12 (25%)	3 (7.9%)	0.038*
Other^d^	4 (8.3%)	2 (5.3%)	0.46 (MoTHer app^b^, phone)

	Metropolitan *n* = 48, *n* (%)	Rural *n* = 38, *n* (%)	Total *n* = 86, *n* (%)
**Impact of COVID‐19 on care delivery** ^**c**^			
Yes	41 (85%)	30 (79%)	0.43
No	7 (15%)	8 (21%)	

	Metropolitan, *n* (%)	Rural, *n* (%)	Total, *n* (%)
**Methods of communicating with multidisciplinary team members** ^**a**^			
Regular meetings/case conferences^c^	13 (27.1%)	17 (44.7%)	0.089
Verbal conversations in clinic^c^	35 (72.9%)	23 (60.5%)	0.22
Emails^c^	35 (72.9%)	30 (78.9%)	0.52
Phone calls^c^	25 (52.1%)	22 (57.9%)	0.59
Letters^c^	15 (31.3%)	17 (44.7%)	0.20
Hospital charts^c^	12 (25%)	15 (39.5%)	0.15
Hand‐held records^d^	7 (14.6%)	4 (10.5%)	0.41
Other^c^—EMR, app^b^	9 (18.8%)	3 (7.9%)	0.15

Abbreviation: EMR, electronic medical record.

^a^ Multiple responses possible.

^b^ Respondents indicated the use of the MoTHer app developed by the CSIRO; trials are currently underway in Queensland.

^c^ Chi squared test of independence.

^d^ Fishers Exact test.

* Indicates significant less than or equal to 0.05.

*** Highly significant less than or equal to 0.001.

Both metropolitan (73%, *n* = 35) and rural (79%, *n* = 30) health professionals used emails most frequently to communicate with other multidisciplinary team members. Verbal conversations in the clinic were more frequently used by metropolitan health professionals (72.9%, *n* = 35) compared to rural (60.5%, *n* = 23). Refer to Table 2 for all responses to the methods of communication used between team members.

Most respondents (83%, *n* = 71) reported that COVID‐19 had impacted their care delivery for people with GDM. Main impacts on care included: an increased use of telehealth (58%, *n* = 56), less face‐to‐face services (13%, *n* = 12), less group education (8%, *n* = 8), changes in methods of diagnosis (6%, *n* = 6) and difficulty in communicating with patients (5%, *n* = 5).

The differences between metropolitan and rural respondents' level of satisfaction with the model of care were not statistically significant (*p*‐value 0.0572) (Appendix S2). There was no statistically significant difference between metropolitan and rural respondents' level of satisfaction with multidisciplinary team communication (*p*‐value 0.203) (Appendix S2).

## 4 Discussion

The current survey is the first to compare rural and metropolitan healthcare delivery for people with DIP across Australia. Overall, many aspects of care were reported to be similar for both metropolitan and rural areas, with the main differences relating to availability of health professionals and mode of care delivery. Current research suggests key considerations for service delivery is that it is integrated, includes a multidisciplinary team approach, is patient centred and flexible to meet the individual service needs [4, 8, 9]. Surveys investigating DIP care in developed countries internationally have largely not investigated rural care delivery [18, 19]. A New Zealand study compared service delivery for GDM between metropolitan and rural professionals and found minimal differences; however, only dietitians were surveyed, with a small sample size of 33 participants [20]. Previous Australian surveys have tended to be state or territory specific [12, 21, 22], and though some national surveys reported the number of metropolitan and rural respondents [3, 11], results were not separated by location.

In this current study, most metropolitan and rural health professionals were satisfied with their model of care and the multidisciplinary team communication used in their workplaces. Although there was no statistically significant difference between metropolitan and rural respondents, there was a trend towards rural health professionals being less satisfied with their current model of care (Appendix S2). Previous Australian surveys have not included a focus on satisfaction with the model of care [3, 11, 13, 22]. However, two surveys conducted in Queensland [21] and the Northern Territory [12] asked about satisfaction with specific aspects of care, including communication [12, 21]. One survey found very low rates of satisfaction with hospital‐related communication (1%–4%) [21], reflecting previous studies indicating hospital communication, particularly discharge summaries back to general practitioners, a key issue [23, 24, 25]. Interestingly, the survey that compared urban and remote responses found no difference in satisfaction rates between these professionals [12], similar to the current survey results.

Communication methods used with patients varied, with similarities in the methods used between metropolitan and rural health professionals. Contrary to previous literature where the uptake of telehealth by rural professionals was limited [26], the current survey found there were similarities with the use of telehealth between rural and metropolitan respondents. This may be due to the rapid uptake of telehealth practices during the COVID‐19 pandemic across the health sector regardless of location [27]. Interestingly, face‐to‐face consults were only offered by 70% of metropolitan respondents compared to 100% of rural respondents, whereas in a 2017 study, 98% of respondents reported offering initial face‐to‐face consults [3]. As the current survey was conducted following the COVID‐19 related increase in telehealth services [27], this may account for the reduction in health professionals providing face‐to‐face services compared to 2017. However, this does not account for the finding that only metropolitan health professionals reported having reduced their use of face‐to‐face services. This lack of face‐to‐face services offered by metropolitan respondents may be due to these professionals transitioning exclusively to telehealth care delivery [28].

Multidisciplinary team communication has been described previously as a key element in DIP care delivery [12, 21, 22]. Issues with multidisciplinary team communication have been associated with disjointed systems that impact health professional care coordination [21, 22]. The current research highlights the importance of multidisciplinary team communication, with the majority of both metropolitan and rural health professionals satisfied with their team communication. Key methods of communication used included emails, verbal conversations in clinic and phone calls. Optimising communication may be a key element to explore further when assessing rural healthcare models and identifying strategies for improvement.

Differences in access to health professionals and services in rural and metropolitan areas have been identified as problematic in Australia [4]. These discrepancies continue, with lower proportions of full time equivalent specialist medical practitioners and allied health professionals available in rural areas compared to metropolitan [5, 29]. In the current study, rural health professionals were less likely to be co‐located and have face‐to‐face communication with obstetricians and endocrinologists compared to those in metropolitan areas. Limited availability of clinicians in rural areas may impact the model of care as well as adherence to diabetes care guidelines. Inadequate access to a multidisciplinary team may lead to increased risk of adverse maternal and birth outcomes [1, 2]. Development and evaluation of flexible models of care may address these issues, which could be a key area of future research.

A main strength of the current survey is the strong rural representation, with 44% from rural areas. Additionally, this survey compares metropolitan and rural healthcare delivery practices including a broad distribution of respondents across different Australian states and territories. A limitation is that the largest proportion of respondents were from two professions: dietitians (*n* = 51) and diabetes educators (*n* = 25), which may be due to response bias from team members who are frequently involved in DIP care. Due to the recruitment strategy focusing on low‐cost methods of dissemination, we were unable to engage with specialist bodies for targeted recruitment. Poor response rates from certain physician specialities, including general practitioners, are a common issue [30]. This may limit the generalisability of the study's results. Another limitation is the response rate was unable to be calculated due to the unknown number of clinicians who were invited to complete the survey. Additionally, the sample size may have limited the ability to obtain statistically significant results.

Metropolitan and rural health professionals reported many similarities in aspects of their DIP care delivery for women with diabetes in pregnancy. Key differences occurred in the offering of face‐to‐face consults and reduced availability of specialist clinicians in rural locations. All health professionals identified multidisciplinary team communication as an essential element within healthcare delivery, which is likely to be a key area of focus when modifying or developing innovative care models designed to optimise outcomes for women with DIP. Additional research is required to further understand the experiences of clinicians delivering DIP healthcare in rural areas. A comprehensive understanding of the competing demands affecting successful DIP health care delivery in the rural context will enable healthcare delivery practices to be developed that address the needs of rural communities.

## Funding

E.P. received an RTP (Research Training Program) Scholarship from the University of Newcastle. S.H. and L.J.B. are employed in roles funded through the Australian Government Department of Health and Aged Care's Rural Health Multidisciplinary Program Funding. C.C. is supported by an NHMRC Research Leader Fellowship (L3, APP2009340).

## Conflicts of Interest

The authors declare no conflicts of interest.

## Supporting information


**Appendix S1:** ajo70109‐sup‐0001‐AppendixS1.pdf.

**Appendix S2:** ajo70109‐sup‐0002‐AppendixS2.docx.

## Data Availability

The datasets used and/or analysed during the current study are available from the corresponding author on reasonable request.
