# Supplementary material for: Healthcare for People With Diabetes in Pregnancy: A National Survey Comparing Metropolitan and Rural Care Delivery in Australia
Source: Aust N Z J Obstet Gynaecol. 2026 Mar 25;66(2):e70109. doi: 10.1111/ajo.70109 (PMC13015180; doi:10.1111/ajo.70109)
Supplement: Supplementary file 1 — Appendix S1: ajo70109‐sup‐0001‐AppendixS1.pdf. [file AJO-66-0-s002.pdf]

This survey asks questions regarding your experiences treating women with diabetes in pregnancy. At certain points in the survey, questions will be asked specifically about treatment for women with gestational diabetes mellitus or pre-existing diabetes in pregnancy (Type 1 or Type 2 diabetes).

SECTION A: Demographic information

The following questions relate to your health professional role where you work women with diabetes in pregnancy.

1 What is the location of your primary place of work?

Street name and suburb ______
Postcode ______

2 What is your professional role in the care of women with diabetes in pregnancy?

- ☐ Diabetes educator or Credentialed diabetes educator
- ☐ Dietitian
- ☐ Endocrinologist/ Diabetes specialist
- ☐ Obstetrician
- ☐ Midwife
- ☐ Nurse
- ☐ General practitioner
- ☐ Other (please specify) ______

3 How many years in total have you worked in your profession?

- ◯ < 1 year
- ◯ 1-4 years
- ◯ 5-10 years
- ◯ 11-20 years
- ◯ 21-30 years
- ◯ >30 years

4 How many years in total have you worked with women with diabetes in pregnancy?

- ◯ < 1 year
- ◯ 1-4 years
- ◯ 5-10 years
- ◯ 11-20 years
- ◯ 21-30 years
- ◯ >30 years

5 Which setting/s best describe where you see women with diabetes in pregnancy? (select all that apply)

- ☐ Public hospital with specialised diabetes in pregnancy services
- ☐ Public hospital without specialised diabetes in pregnancy services
- ☐ Public diabetes centre
- ☐ Private hospital
- ☐ Private practice
- ☐ Community health centre
- ☐ Other (please specify) ______

6 How many women on average would you see with diabetes in pregnancy each month (initial appointment and reviews)?

- ◯ 5 or fewer
- ◯ 6 - 10
- ◯ 11 - 20
- ◯ More than 20

SECTION B: The management of women with gestational diabetes melllitus
The following questions relate to the management and treatment of women with Gestational Diabetes Mellitus at your primary place of work.

7 Does your workplace/ health service use guidelines for the management and treatment of women with GDM?

- ◯ Yes (please specify the guidelines used in the comment box below e.g. ADIPS, RACGP) ______
- ◯ Yes, but I use my professional expertise to guide the way I manage and treat women with GDM
- ◯ No
- ◯ Unsure

8 After diagnosis, which health care professional(s) do women see for initial education and advice on managing GDM and what format does this take? (select all that apply)

	Group education	Individual appointment	Unsure	Not applicable
Diabetes educator or Credentialed diabetes educator	◯	◯	◯	◯
Dietitian	◯	◯	◯	◯
Endocrinologist/ Diabetes specialist	◯	◯	◯	◯
Obstetrician	◯	◯	◯	◯
Midwife	◯	◯	◯	◯
Nurse	◯	◯	◯	◯
General practitioner	◯	◯	◯	◯
Other (please specify) ______	◯	◯	◯	◯

9 How soon after referral are women with GDM usually seen for initial education?

- ◯ < 1 week
- ◯ 1 -2 weeks
- ◯ 3 - 4 weeks
- ◯ >1 month
- ◯ Unsure

10 What mode of initial education does your service provide for women with GDM? (select all that apply)

- ☐ Face to face
- ☐ Telehealth
- ☐ Written information via post, email or fax
- ☐ Video presentation
- ☐ Other (please specify) ______

11 What does the initial approach for managing GDM involve at your clinic/ service? (select all that apply)

- ☐ General education on GDM and its management e.g. what is GDM, short and long term impacts
- ☐ Medical Nutrition Therapy (specialised dietary advice) as provided by a dietitian
- ☐ Diet advice provided by a non-dietitian health care professional or medical specialist
- ☐ Physical activity advice
- ☐ Self-blood glucose monitoring advice
- ☐ Gestational weight gain advice
- ☐ Commencement of pharmacotherapy (insulin or oral hyperglycaemic agent)
- ☐ Other (please specify) ______

The following question/s relate to treatment and management of women with gestational diabetes mellitus after the initial education/ appointment.

12 After initial education, who do women usually see for follow up? (select all that apply)

- ☐ Diabetes educator or Credentialed diabetes educator
- ☐ Dietitian
- ☐ Endocrinologist/ Diabetes specialist
- ☐ Obstetrician
- ☐ Midwife
- ☐ Nurse
- ☐ General practitioner
- ☐ Unsure
- ☐ Other (please specify) ______

13 After initial education, on average how often are women with GDM seen by each health care professional?

	Weekly	Fortnightly	Monthly	Less than monthly	Unsure	Not seen/ Not applicable
Diabetes educator/ Credentialed diabetes educator	○	○	○	○	○	○
Dietitian	○	○	○	○	○	○
Endocrinologist/ Diabetes specialist	○	○	○	○	○	○
Obstetrician	○	○	○	○	○	○
Midwife	○	○	○	○	○	○
Nurse	○	○	○	○	○	○
General practitioner	○	○	○	○	○	○
Other (please specify) ______	○	○	○	○	○	○

The following question/s relate to ongoing monitoring in the management of women with gestational diabetes mellitus.

14 Which blood glucose measures does your workplace/ health service use for ongoing monitoring? (select all that apply)

- ☐ Fasting
- ☐ 1 hour post prandial
- ☐ 2 hour post prandial
- ☐ Other (please specify) ______

15 What are the capillary blood glucose targets given to women who are not on any form of pharmacotherapy?

Note:

Queensland Clinical Guideline cut offs - fasting ≤5 mmol/L, 1 hour postprandially ≤7.4 mmol/L, 2 hour postprandially ≤6.7 mmol/L

RACGP Guideline cut offs - fasting between 4 and 6 mmol/ L or 2 hours postprandially: ≤7mmol/L

- ◯ Queensland Clinical Guidelines/ ADIPS
- ◯ RACGP Guidelines
- ◯ Unsure
- ◯ Other (please specify) ______

16 Who is responsible for the ongoing review of BGLs? (Select all that apply)

- ☐ Diabetes educator or Credentialed diabetes educator
- ☐ Dietitian
- ☐ Endocrinologist/ Diabetes specialist
- ☐ Obstetrician
- ☐ Midwife
- ☐ Nurse
- ☐ General practitioner
- ☐ Unsure
- ☐ Other (please specify) ______

17 Is gestational weight gain tracked throughout a GDM pregnancy?

- ◯ Yes
- ◯ No
- ◯ Unsure

17 a) If yes, which health professional/s are responsible in your place of work for tracking gestational weight gain throughout a GDM pregnancy? (select all that apply)

- ☐ Diabetes educator or Credentialed diabetes educator
- ☐ Dietitian
- ☐ Endocrinologist/ Diabetes specialist
- ☐ Obstetrician
- ☐ Midwife
- ☐ Nurse
- ☐ General practitioner
- ☐ Unsure
- ☐ Other (please specify) ______

18 For women who are managing their GDM through diet only, who is primarily responsible for their ongoing GDM management?

- ◯ Diabetes educator or Credentialed diabetes educator
- ◯ Dietitian
- ◯ Endocrinologist/ Diabetes specialist
- ◯ Obstetrician
- ◯ Midwife
- ◯ Nurse
- ◯ General practitioner
- ◯ Unsure
- ◯ Other (please specify) ______

19 For women who are managing their GDM through diet only, what factors would warrant ongoing dietary advice? (select all that apply)

- ☐ Excess gestational weight gain
- ☐ Weight loss
- ☐ Patient concerns about nutrient deficiencies
- ☐ Health care professional's concerns about nutrient deficiencies
- ☐ Unstable BGLs
- ☐ Unsure
- ☐ Other (please specify) ______

20 If hyperglycaemia is present at follow up, which strategy(ies) are ideally used before making decisions about further treatment? (select all that apply)

- ☐ Review of food diary or food intake
- ☐ Re-education on dietary management including more prescriptive dietary advice where appropriate
- ☐ Tracking gestational weight gain
- ☐ Foetal surveillance/ foetal growth
- ☐ Review of self-blood glucose monitoring techniques and timing
- ☐ Unsure
- ☐ Other (please specify) ______

21 Who is responsible for identifying women who need additional intervention beyond diet therapy? (select all that apply)

- ☐ Diabetes educator or Credentialed diabetes educator
- ☐ Dietitian
- ☐ Endocrinologist/ Diabetes specialist
- ☐ Obstetrician
- ☐ Midwife
- ☐ Nurse
- ☐ General practitioner
- ☐ Unsure
- ☐ Other (please specify) ______

The following question/s relate to pharmacotherapy practices in the management of women with Gestational Diabetes Mellitus.

22 Please provide a description of how women with GDM are identified as requiring pharmacotherapy commencement at your service.

Decisions regarding when a woman should commence pharmacotherapy often vary depending on the health service or decision-maker. There are a number of different examples provided below but this might be different from what happens at your workplace.

Examples:
The average BGL over 1 week is elevated (BGL at the same time each day)
BGL are elevated on 2 or more occasions at the same test point within 1 week
Fasting BGLs are consistently above target
There is more than one BGL above target that cannot be explained by diet
Elevated BGL and evidence of fetal macrosomia
Based on a computer algorithm/ program

Please provide as much detail as you require in the space below.

---

23 What is used as first line pharmacotherapy at your service?

- ◯ Insulin
- ◯ Metformin
- ◯ Unsure
- ◯ Other (please specify) ______

---

24 What is used as second line pharmacotherapy at your service?

- ◯ Only first line pharmacotherapy agent is used
- ◯ Insulin
- ◯ Metformin
- ◯ Unsure
- ◯ Other (please specify) ______

---

25 Once a woman commences pharmacotherapy, do the capillary blood glucose targets remain the same?

- ◯ Yes
- ◯ No (please provide more detail in the box below) ______
- ◯ Unsure

The following question/s relate to postpartum management of women with Gestational Diabetes Mellitus.

26	Is advice given to women about postpartum management and follow-up? If so, what advice is given? (select all that apply)	☐ Information on postpartum testing<br>☐ Advice to optimise postpartum and inter-pregnancy weight<br>☐ Dietary or other lifestyle advice<br>☐ Breastfeeding advice<br>☐ Unsure<br>☐ No information routinely given<br>☐ Other (please specify) ______
27	Who is responsible for conducting postpartum testing for women?	◯ GP appointment for postpartum testing (for example OGTT at 6 - 12 weeks)<br>◯ Hospital appointment for postpartum testing (for example OGTT at 6 - 12 weeks)<br>◯ Unsure<br>◯ Other (please specify) ______

The following question/s relate to the management of pregnant women with pre-existing diabetes (Type 1 and Type 2 diabetes).

28 For women with pre-existing diabetes in pregnancy (Type 1 and Type 2 diabetes) does your workplace have a separate clinic?

- ◯ Yes
- ◯ No
- ◯ Unsure

28 a) Please provide a brief description of the typical service for women with pre-existing diabetes in pregnancy (i.e. how and when are women seen, how are they typically managed?)

____

SECTION C: Multidisciplinary team care coordination

The following questions relate to the multidisciplinary care coordination for women with diabetes in pregnancy

29 Please indicate which members of the multidisciplinary team (MDT) are available in your clinic/service for women with diabetes in pregnancy (select all that apply):

	Yes, co-located at same clinic/ service	Yes, at a separate local address	Yes, via telehealth	Not available/ unsure
Diabetes educator/ Credentialed diabetes educator	○	○	○	○
Dietitian	○	○	○	○
Endocrinologist/ Diabetes specialist	○	○	○	○
Obstetrician	○	○	○	○
Midwife	○	○	○	○
Nurse	○	○	○	○
General practitioner	○	○	○	○
Exercise physiologist/ physiotherapist	○	○	○	○
Lactation consultant	○	○	○	○
Aboriginal Health worker/ Aboriginal Health Practitioner	○	○	○	○
Mental health professional e.g. psychologist, mental health nurse	○	○	○	○
Other (please specify) ______	○	○	○	○

30 Do you deliver services jointly with any other members of the MDT?

	Yes, initial education in a group	Yes, individual initial education	Yes, follow up in a group	Yes, individual follow up	No, I don't jointly deliver services with this team member/ Not applicable
Diabetes educator/ Credentialed diabetes educator	☐	☐	☐	☐	☐
Dietitian	☐	☐	☐	☐	☐
Endocrinologist/ Diabetes specialist	☐	☐	☐	☐	☐
Obstetrician	☐	☐	☐	☐	☐
Midwife	☐	☐	☐	☐	☐

Nurse	☐	☐	☐	☐	☐
General practitioner	☐	☐	☐	☐	☐
Lactation consultant	☐	☐	☐	☐	☐
Aboriginal Health Worker/ Aboriginal Health Practitioner	☐	☐	☐	☐	☐
Mental health professional e.g. psychologist, mental health nurse	☐	☐	☐	☐	☐
Other (please specify) ______	☐	☐	☐	☐	☐

31 How do you communicate with MDT members about specific patients? (select all that apply)

- ☐ Regular meetings/ case conferences
- ☐ Verbal conversations in clinic
- ☐ Emails
- ☐ Phone calls
- ☐ Letters
- ☐ Hospital charts
- ☐ Hand-held records
- ☐ Other (please specify) ______

32 How satisfied are you with your current location's overall model of care?

- ◯ Very satisfied
- ◯ Somewhat satisfied
- ◯ Neutral
- ◯ Somewhat unsatisfied
- ◯ Very unsatisfied

32 a)If very satisfied, what works well?

____

32 a)What improvements would you make?

____

32 b)What do you see as the barriers to making these improvements at your service location?

____

33 How satisfied are you with the frequency and quality of communication with other MDT members?

- ◯ Very satisfied
- ◯ Somewhat satisfied
- ◯ Neutral
- ◯ Somewhat unsatisfied
- ◯ Very unsatisfied

33 a)If very satisfied, what works well?

____

33 a)What improvements would you make?

____

33 b)What do you see as the barriers to making these improvements at your service location?

____

SECTION D: GDM management in the context of COVID-19
The following question/s are about the impact COVID-19 has had on care delivery for women with Gestational Diabetes Mellitus.

34 Has COVID-19 affected your care delivery to women with GDM ?

◯ Yes
◯ No
◯ Unsure

34 a) If Yes, please provide additional details

____

34 b) If you have made changes to the care delivery are any of these likely to remain? Please explain.

____

Once you press the 'submit' button below, you will not be able to change your answers or remove your anonymous responses from the study.
