# Supplementary material for: Healthcare for People With Diabetes in Pregnancy: A National Survey Comparing Metropolitan and Rural Care Delivery in Australia
Source: Aust N Z J Obstet Gynaecol. 2026 Mar 25;66(2):e70109. doi: 10.1111/ajo.70109 (PMC13015180; doi:10.1111/ajo.70109)
Supplement: Supplementary file 2 — Appendix S2: ajo70109‐sup‐0002‐AppendixS2.docx. [file AJO-66-0-s001.docx]

**Appendix S2: Health professional ratings of level of satisfaction with model of care in work location (rural vs metropolitan)**

| **Model of care satisfaction (n=86)** | | | | | |
| --- | --- | --- | --- | --- | --- |
| Metropolitan  (MMM1)  Rural (MMM2-7) | **Very satisfied** | **Somewhat satisfied** | **Neutral** | **Somewhat unsatisfied** | **Very unsatisfied** |
| **MMM1** | 15 (31.3%) | 24 (50%) | 3 (6.3%) | 5 (10.4%) | 1 (2.1%) |
| **MMM2-7** | 6 (15.8%) | 20 (52.6%) | 1 (2.6%) | 8 (21.1%) | 3 (7.9%) |
| ***Communication* *satisfaction* (n=86)** | | | | | |
| **MMM1** | 25 (52.1%) | 16 (33.3%) | 3 (6.3%) | 3 (6.3%) | 1 (2.1%) |
| **MMM2-7** | 11 (28.9%) | 15 (39.5%) | 6 (15.8%) | 4 (10.5%) | 1 (2.6%) |
